# Effect of Paternal Genome Excess on the Developmental and Gene Expression Profiles of Polyspermic Zygotes in Rice

**DOI:** 10.3390/plants10020255

**Published:** 2021-01-28

**Authors:** Ryouya Deushi, Erika Toda, Shizuka Koshimizu, Kentaro Yano, Takashi Okamoto

**Affiliations:** 1 Department of Biological Sciences, Tokyo Metropolitan University, Tokyo 192-0392, Japan; underworld0326@gmail.com (R.D.); etoda@tmu.ac.jp (E.T.); 2 Department of Life Sciences, Meiji University, Kanagawa 214-8571, Japan; ty18004@meiji.ac.jp (S.K.); kyano@meiji.ac.jp (K.Y.)

**Keywords:** fertilization, male excess, parental genome, paternal genome, polyspermy, rice

## Abstract

Polyploid zygotes with a paternal gamete/genome excess exhibit arrested development, whereas polyploid zygotes with a maternal excess develop normally. These observations indicate that paternal and maternal genomes synergistically influence zygote development via distinct functions. In this study, to clarify how paternal genome excess affects zygotic development, the developmental and gene expression profiles of polyspermic rice zygotes were analyzed. The results indicated that polyspermic zygotes were mostly arrested at the one-cell stage after karyogamy had completed. Through comparison of transcriptomes between polyspermic zygotes and diploid zygotes, 36 and 43 genes with up-regulated and down-regulated expression levels, respectively, were identified in the polyspermic zygotes relative to the corresponding expression in the diploid zygotes. Notably, *OsASGR-BBML1*, which encodes an AP2 transcription factor possibly involved in initiating rice zygote development, was expressed at a much lower level in the polyspermic zygotes than in the diploid zygotes.

## 1. Introduction

Fertilization is a characteristic event of eukaryotic unicellular and multicellular organisms that combines male and female genetic materials for the next generation. In the diploid zygote generated by the fusion between haploid male and female gametes, parental genomes function synergistically to ensure the faithful progression of zygotic development and the subsequent embryogenesis. In angiosperms, sporophytic generation is initiated by a double fertilization to form seeds that are consisting of three tissues, embryo, endosperm and maternal seed coat [1]. Regarding the double fertilization, one sperm cell fuses with the egg cell, resulting in the formation of a zygote, and another sperm cell fuses with the central cell to form a triploid primary endosperm cell. The zygote and primary endosperm cell respectively develop into the embryo, which carries genetic material from the parents, and the endosperm, which nourishes the developing embryo and seedling [2,3,4]. Of the three tissues in seeds, it has been known that the endosperm is highly sensitive to an imbalanced parental genome ratio resulting from ploidy differences between the parents [5,6,7,8,9]. 

In a recent study, the effects of parental genome imbalance on zygotic development were clarified by producing polyploid zygotes with an imbalanced parental genome ratio via the *in vitro* fertilization of isolated rice gametes and by elucidating the developmental profiles of the polyploid zygotes [10,11]. The results indicated that approximately 50%–75% of the polyploid zygotes with an excess of paternal genome content exhibited the developmental arrest, whereas most of the polyploid zygotes with an excess of maternal gamete/genome content developed normally, as diploid zygotes [10]. Notably, the paternal excess zygotes did not progress beyond the first zygotic division, although karyogamy was completed normally. These results suggest that parental genomes have different functions and are used synergistically in zygotes. Moreover, the early zygotic developmental steps, from karyogamy to the first cell division, are highly sensitive to paternal genome excess. Consistent with the possible preferential functions of parental genomes in zygotic embryogenesis, genes expressed in a monoallelic and/or parent-of-origin manner during zygotic development and/or early embryogenesis have been identified, and the functions of some monoallelic genes during early embryogenesis have been thoroughly investigated [12,13,14,15,16,17,18]. In addition, it has been reported that genes relating to cell cycle, RNA processing, signaling pathway and other cellular machineries are involved in zygotic division and/or development [19,20,21,22,23,24,25,26]. However, it remains unclear how parental genomes function synergistically in developing zygotes.

In the present study, we focused on the developmental characteristics of paternal excess rice zygotes (i.e., polyspermic zygotes), since the developmental arrest of the polyspermic zygote would be due to the excess male genomic content in the nucleus, wherein the imbalanced parental genomes may adversely affect zygotic development. The possible mechanism underlying the dysfunction between parental genomes is partly clarified by comparing the developmental and gene expression profiles of the polyspermic zygotes with those of diploid zygotes [10]. Therefore, development of polyspermic rice zygote was carefully monitored to identify the stage in which the developmental arrest becomes evident. Furthermore, the transcriptomes of the polyspermic zygotes and diploid zygotes were compared to determine the effects of the paternal excess on the zygote gene expression profiles.

## 2. Results

### 2.1. Developmental Profiles of Polyspermic Rice Zygotes

**Figure 1:**
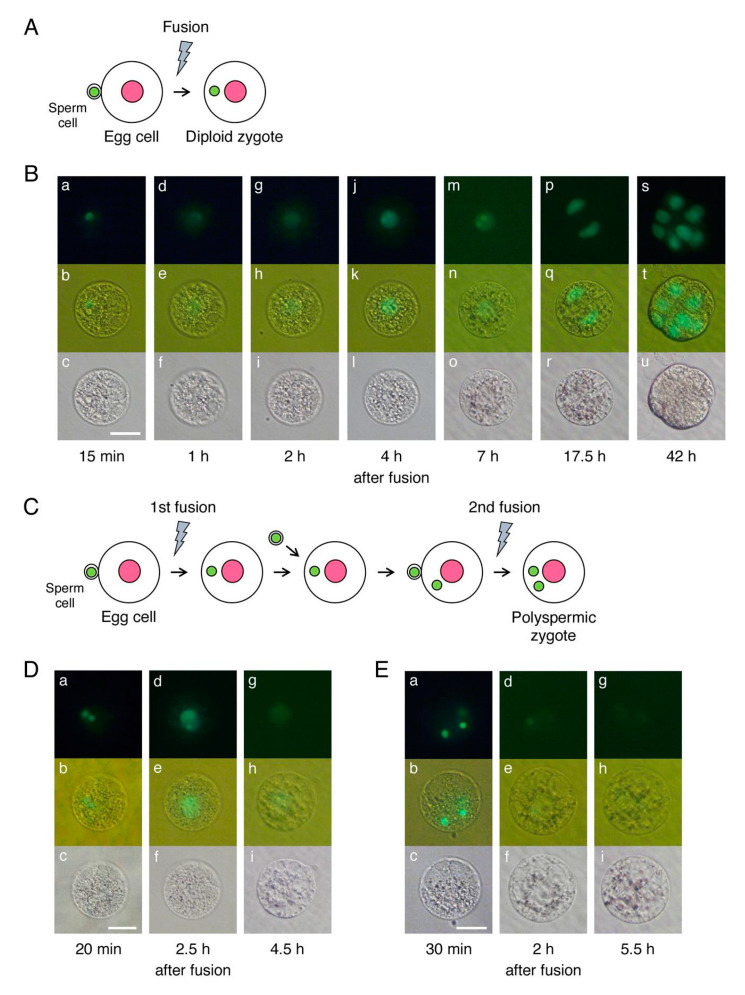
Developmental profiles of a diploid zygote (**A**,**B**) and polyspermic zygotes (**C**–**E**). (**A**) Schematic illustration of the production of diploid rice zygotes. An egg cell and a sperm cell were fused to produce a monospermic diploid zygote. (**B**) Developmental profiles of a diploid rice zygote. A sperm nucleus fluorescently labeled with H2B-GFP was observed in the zygote (a–c) and karyogamy progressed in the zygote (d–l). Thereafter, the zygote developed into a two-celled embryo (m–r) and a globular-like embryo (s–u). (**C**) Schematic illustration of the production of polyspermic rice zygotes. Two sperm cells were sequentially fused to an egg cell to produce a polyspermic zygote as described by Toda et al. (2016) [28]. (**D**) Progression of karyogamy in polyspermic zygotes. Two sperm nuclei fluorescently labeled with H2B-GFP were detected in the polyspermic zygote at 20 min after the fusion (a–c). The two nuclei then fused with an egg nucleus, resulting in a detectable zygotic nucleus (d–i). (**E**) Lack of karyogamy in polyspermic zygotes. Although two sperm nuclei fluorescently labeled with H2B-GFP were observed in the fused egg cell (a–c), the progression of karyogamy was undetectable (d–i). Pink and green circles in (**A**,**C**) indicate the egg and sperm nuclei, respectively. The gray flash symbols in (**A**,**C**) represent electro-fusions. Top, middle, and bottom panels in (**B**,**D**,**E**) represent fluorescent, merged fluorescent/bright-field, and bright-field images, respectively. Scale bars = 20 µm.

In this study, sperm cells isolated from transformed rice plants expressing histone H2B-GFP were used to produce zygotes for the subsequent visualization of the nucleus in developing zygotes. Diploid zygotes were produced via the electro-fusion between egg and sperm cells (Figure 1A). The zygotes developed into a two-celled embryo at 17.5 h after gamete fusion and a globular-like embryo was formed via repeated cell division at 42 h after gamete fusion (Figure 1B) [27]. Polyspermic zygotes were generated using one egg cell and two sperm cells (Figure 1C) [28]. We produced 34 polyspermic zygotes for the sequential monitoring of developmental steps from karyogamy to the first zygotic division. In an earlier study, we analyzed the developmental profiles of polyspermic zygotes daily after the gametes fused to ascertain whether the cells of the polyspermic zygotes were dividing [10], and were unsuccessful in determining exactly when the degeneration of developing polyspermic zygotes becomes apparent.

**Figure 2:**
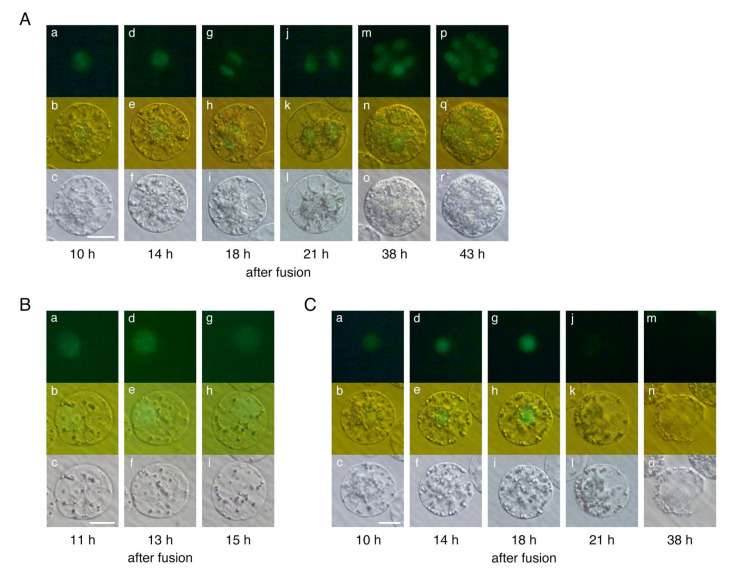
Developmental profiles of polyspermic rice zygotes after karyogamy. An egg cell was serially fused with two sperm cells expressing H2B-GFP, and the resulting zygote was analyzed. (**A**) After karyogamy, the polyspermic zygotes developed and divided into a two-celled embryo (a–l) and a globular-like embryo (m–r). (**B**) Developmental arrest of polyspermic zygotes (pattern I). Although the H2B-GFP signal was detectable in the zygotic nucleus, zygotes were highly vacuolated and became transparent (a–i) before they degenerated. (**C**) Developmental arrest of polyspermic zygotes (pattern II). The H2B-GFP signal was clearly detected in the zygotic nucleus during development (a–i); however, the fluorescent signal decreased and was undetectable at approximately 21 h after the fusion (j–l). The zygotes degenerated without dividing (m–o). Top, middle, and bottom panels represent fluorescent, merged fluorescent/bright-field, and bright-field images, respectively. Scale bars = 20 µm.

**Figure 3:**
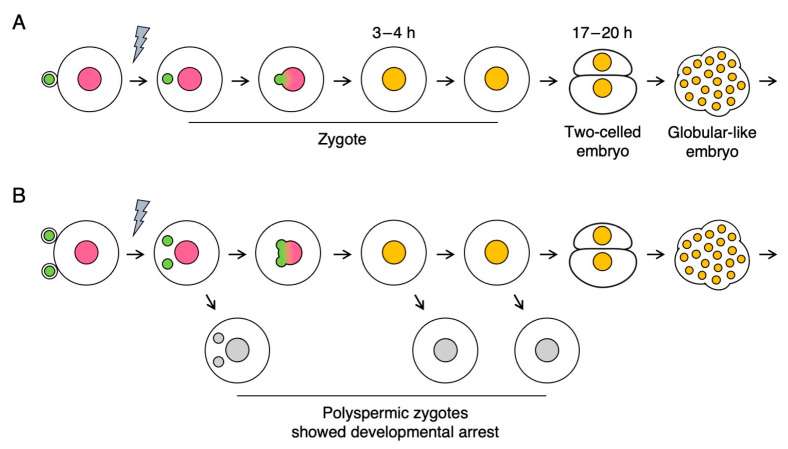
Schematic diagram of the early development of the diploid zygote (**A**) and polyspermic zygote (**B**). The times required for the completion of karyogamy (ca. 3–4 h) and the first cell division (ca. 17–20 h) are provided. Pink, green, and orange circles indicate the egg, sperm, and zygotic nuclei, respectively. Gray circles indicate the egg, sperm, and zygotic nuclei in the polyspermic zygotes that exhibited arrested development. The gray flash symbols represent electro-fusions.

Among the 34 polyspermic zygotes, karyogamy, which involves the fusion of two sperm and one egg nuclei to form a zygotic nucleus, was detected in 30 zygotes (Figure 1D; Table 1). Karyogamy was undetectable in the other four polyspermic zygotes (Figure 1E), which subsequently degenerated. Upon the completion of karyogamy, 19 of the 30 polyspermic zygotes divided into two-celled and globular-like embryos (Figure 2A) similar to diploid zygotes (Figure 1B). Arrested development was observed in the remaining 11 polyspermic zygotes (Table 1), suggesting that approximately one-third of the polyspermic zygotes were affected by post-karyogamy defects during development. This tendency was consistent with the results of our previous analysis of the cell division profiles of polyspermic zygotes (Supplemental Table S1) [10]. The developmental profiles of the 11 polyspermic zygotes after karyogamy revealed two degeneration patterns. Specifically, for nine of the polyspermic zygotes, the cells became transparent and appeared to be highly vacuolated at approximately 11–15 h after gamete fusion (Figure 2B). Additionally, the intensity of the fluorescent signals from the H2B-GFP in the nucleus decreased to low levels (Figure 2B), and the zygotes finally degenerated. This degeneration pattern was considered to reflect the main developmental defects of polyspermic zygotes. Regarding the other two polyspermic zygotes, abnormal cellular characteristics were not evident at approximately 10–18 h after the fusion (Figure 2C), and the fluorescence intensity in the nucleus was equivalent to that of diploid and/or polyspermic zygotes which divided into two-celled embryos (Figure 1B, Figure 2A,C). However, the fluorescent signals in the nucleus of these two polyspermic zygotes became undetectable at approximately 21 h after the fusion (Figure 2C), which is just before the first zygotic division. The zygotes then degenerated without dividing (Figure 2C). These two types of degeneration profiles suggest that developmental defects can be triggered at early and late developmental stages (Figure 3), and that the early developmental stage, probably after karyogamy, is primarily when zygotic development is affected by imbalanced parental genomes. Therefore, polyspermic zygotes and diploid zygotes at 4–5 h after gamete fusion (i.e., following the completion of karyogamy) were freshly prepared for transcriptome analyses.

### 2.2. Gene Expression Profiles of Polyspermic Zygotes

**Figure 4:**
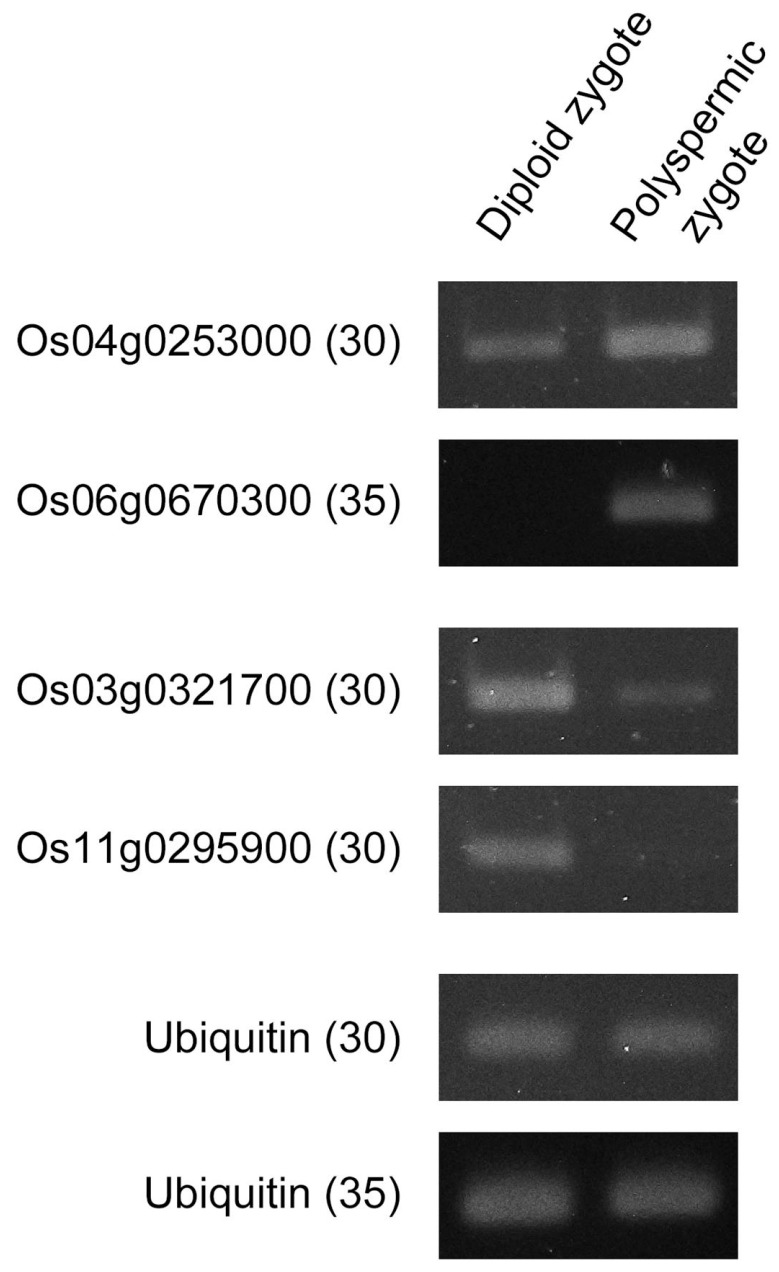
Expression patterns of 4 genes whose expression levels were putatively up- or down-regulated in polyspermic zygotes. Semi-quantitative RT-PCR was performed on cDNAs synthesized from diploid and polyspermic zygotes using specific primers for the putatively up-regulated genes, Os04g0253000 and Os06g0670300 (Table 2) and down-regulated genes, Os03g0321700 and Os11g0295900 (Table 3) in polyspermic zygotes. Ubiquitin cDNA was used as an internal control. Numbers in parentheses indicate the number of PCR cycles. Primer sequences are presented in Supplementary Table S5.

To identify genes with misregulated expression in polyspermic zygotes, the differentially expressed genes (DEGs) between polyspermic and diploid zygotes were analyzed. Relative to the corresponding expression in the diploid zygotes, 36 and 43 genes with up-regulated and down-regulated expression levels, respectively, were identified in the polyspermic zygotes (Table 2 and Table 3, Supplemental Tables S2 and S3). Expression profiles of the representative 4 up- or down-regulated genes in polyspermic zygotes were confirmed using semi-quantitative RT-PCR (Figure 4). The enriched gene ontology (GO) terms among the up-regulated genes in the polyspermic zygotes were related to chromatin/chromosomal assembly/organization (Supplemental Table S4). Whereas, no GO term was enriched among the down-regulated genes.

**Figure 5:**
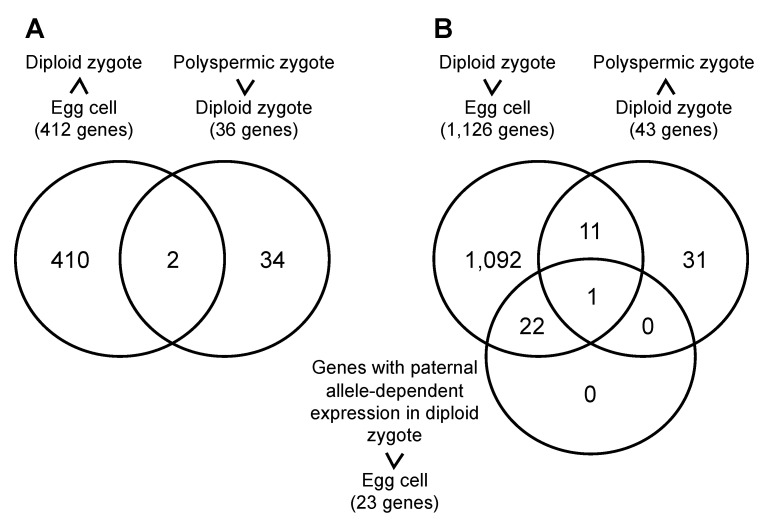
Gene expression in rice polyspermic zygotes and diploid zygotes. (**A**) Venn diagram of 412 genes, whose expression levels in diploid zygotes are suppressed after fertilization [29], and 36 genes, whose expressions are up-regulated in polyspermic zygotes relative to diploid zygotes (Table 2). (**B**) Venn diagram of 1,126 genes, which were detected as fertilization-induced genes in rice diploid zygotes [29], 43 genes, whose expressions are down-regulated in polyspermic zygotes relative to diploid zygotes (Table 3), and 23 genes which are up-regulated in diploid zygotes after fertilization with paternal allele dependent expression [29].

To analyze mis-expressed genes in polyspermic zygotes, gene expression profiles were compared between genes down-regulated in diploid zygotes after fertilization [29] and those up-regulated in polyspermic zygotes relative to diploid zygotes (Table 2). Two genes, Os01g0760000 and Os09g0551600, were identified as overlapped genes (Figure 5A), and, interestingly, Os09g0551600 encoded nucleasome/chromatin assembly factor D protein of HMG protein family. Next, comparison of gene expression profiles was conducted among genes up-regulated in diploid zygotes after fertilization [29], genes down-regulated in polyspermic zygotes relative to diploid zygotes (Table 3) and genes up-regulated in diploid zygotes after fertilization with paternal allele dependent expression [29]. Only one gene, Os11g0295900, was detected in diagram area overlapped with three gene groups (Figure 5B). Notably, the gene encoded *Oryza sativa Apospory-specific Genome Region (ASGR)-BABY-BOOM LIKE (BBML) 1* (*OsASGR-BBML1*) (Table 3), which is a possible initiation factor that is important for zygotic development [29,30].

## 3. Discussion

Paternal genome excess appears to adversely affect polyspermic zygote development mainly during or after the completion of karyogamy. Interestingly, global *de novo* gene expression, termed zygotic genome activation (ZGA), is initiated in rice zygotes during or immediately after karyogamy is completed [31]. Thus, the developmental dysfunction of polyspermic zygotes was predicted to be due to the misexpression of genes important for zygotic development. In addition to gene expression profiles, chromatin/chromosome organization is also considered to be closely associated with plant cell developmental properties [32,33,34]. In a recent study involving chromatin conformation capture (3C) and high-throughput 3C (Hi-C) assays, Zhou et al. (2019) indicated that three-dimensional (3D) genomes of rice egg cells contain a compact silent center (CSC), and that the CSC appears to be reorganized after fertilization and the CSC reorganization may be involved in the regulation of ZGA [35]. The double dose of male chromatin in the nucleus of polyspermic zygotes may affect the 3D genome structure, resulting in abnormal ZGA. Notably, the genes that were more highly expressed in polyspermic rice zygotes than in diploid zygotes were enriched with molecular functions related to chromatin/chromosome assembly/organization (Table 2, Supplemental Table S2). In particular, expression level of a gene Os09g0551600, encoding nucleasome/chromatin assembly factor D protein of HMG protein family, appeared to be extremely high in polyspermic zygote compared to diploid zygotes, in which its expression level is suppressed after gamete fusion. The production of the molecular components required for chromatin/chromosome assembly/organization may be abnormally increased in polyspermic zygotes because of the 3D structure of the paternal excess genome content resulting from the double dose of the male genome. In addition, alternation of genome modification, including DNA methylation and histone acetylation/methylation, in polyspermic zygotes may be a reason for their developmental arrest, since epigenetic reprogramming is supposed to occur during development of zygotes [36,37].

In our previous study for investigating synergistic function of parental genomes in rice zygotes, 23 genes that were preferentially expressed from paternal allele were identified, and it was suggested that monoallelic or preferential gene expression from the paternal genome in the zygote is a safety mechanism for the egg cell, allowing it to suppress the gene expression cascade toward embryogenesis that is normally triggered by fusion with a sperm cell [29]. Therefore, we examined whether misexpression of these 23 genes in polyspermic zygotes occurs or not (Figure 5B). The results indicated that expression level of *OsASGR-BBML1* is highly suppressed in polyspermic zygotes relative to diploid zygotes. *OsASGR-BBML1*, which is alternatively named *OsBBML1*, encodes an AP2 transcription factor that is expressed in a paternal allele-dependent manner in rice zygotes to initiate zygotic development [29,30]. However, the *OsASGR-BBML1* expression level was substantially lower in polyspermic zygotes than in diploid zygotes. Interestingly, it has been reported that the suppression of the OsASGR-BBML1 function in rice zygotes via the ectopic expression of the OsASGR-BBML1-SRDX dominant repressor resulted in the developmental arrest of diploid zygotes at the one-cell stage [29]. The expression of *OsASGR-BBML1* at low levels may result in dysfunctional polyspermic zygotes after karyogamy. The BBM-related transcription factors, including pearl millet *ASGR-BBML1* (*PsASGR-BBML1*) [38,39] and *Brassica napus BBM* (*BnBBM*) [40], reportedly function as determinant factors affecting diverse developmental events in embryonic tissues/cells (e.g., somatic embryogenesis and parthenogenesis). Therefore, elucidating the gene expression cascade triggered by OsASGR-BBML1 in rice zygotes is critical for characterizing the mechanism underlying global embryonic properties as well as zygotic development. Investigations aimed at identifying the genes regulated by OsASGR-BBML1 are currently in progress in our laboratories. 

## 4. Materials and Methods

### 4.1. Plant Materials and Gamete Isolation

*Oryza sativa* L. cv. Nipponbare was grown in an environmental chamber (K30-7248; Koito Industries, Yokohama, Japan) at 26 °C with a 13-h light/11-h dark photoperiod. Transformed rice plants expressing the histone H2B-GFP fusion protein were generated as previously described [41]. Egg cells and sperm cells were isolated from rice flowers using a published procedure [42].

### 4.2. Production and Culture of Diploid Zygotes and Paternal Excess Polyspermic Zygotes

Zygotes were prepared from gametes isolated from wild-type rice plants or transgenic rice plants expressing H2B-GFP. To prepare diploid zygotes, an isolated egg cell and a sperm cell were electro-fused as described [27]. Polyspermic zygotes were produced via a serial fusion between two sperm cells and an egg cell, as previously described [28]. The produced zygotes were cultured on a Millicell-CM insert (Merck KGaA, Darmstadt, Germany) as described [27,43].

### 4.3. Microscopic Analysis

Gametes, zygotes, and embryo-like structures were examined using the BX-71 inverted microscope (Olympus, Tokyo, Japan). The fluorescence of H2B-GFP proteins in cells was observed using the BX-71 inverted fluorescence microscope (Olympus) with 460–490 nm excitation and 510–550 nm emission wavelengths (U-MWIBA2 mirror unit; Olympus). Digital images of the gametes, zygotes, and their cell masses were obtained using a cooled charge-coupled device camera (Penguin 600CL; Pixcera, Los Gatos, CA, USA) and the InStudio software (Pixcera).

### 4.4. cDNA Synthesis, Library Preparation, and mRNA Sequencing

Diploid and polyspermic zygotes cultured for 4–5 h after gamete fusion were washed four times by transferring the cells into fresh droplets of mannitol solution adjusted to 450 mOsmol kg^−1^ H_2_O on coverslips. Each zygote was then transferred into the lysis buffer supplied in the SMART-Seq HT Kit (Takara Bio, Shiga, Japan), after which the lysates were stored at −80 °C until used. cDNA was synthesized and amplified from the cell lysates using the SMART-Seq HT Kit (Takara Bio) according to the manufacturer’s instructions. The resulting amplified cDNA was purified using the Agencourt AMPure XP beads kit (Beckman Coulter, Brea, CA, USA). The quality and quantity of the purified cDNA were determined by the Qubit 3 Fluorometer with a Qubit dsDNA HS Assay Kit (Thermo Scientific, Waltham, MA, USA) and the Agilent 2100 BioAnalyzer with a High Sensitivity DNA chip (Agilent Technologies, Santa Clara, CA, USA). Sequencing libraries were prepared from the amplified cDNA using the Nextera XT DNA Library Prep Kit (Illumina, San Diego, CA, USA), after which they were purified with the Agencourt AMPure XP beads kit. After verifying the quality and quantity of the purified libraries with the Qubit 3 Fluorometer and the Agilent 2100 BioAnalyzer, the libraries were sequenced on the Illumina HiSeqX platform (Illumina) at Macrogen-Japan (Kyoto, Japan) to produce 150-bp paired-end reads.

### 4.5. Analyses of Transcriptome Data

The quality of the Illumina reads was evaluated using FastQC [44]. Regarding the preprocessing of the reads, adapter, poly-A, and low-quality sequences were removed using Cutadapt [45]. The remaining high-quality reads were mapped to the Nipponbare transcript sequences available in RAP-DB [46,47] using RSEM [48] and Bowtie2 [49]. On the basis of the mapping data, the reads mapped to each transcript (TPM) were counted, after which the read count was converted to transcripts per million using RSEM.

The DEGs between the diploid and polyspermic zygotes were identified using TCC [50] of the R software. The number of reads mapped to each transcript was compared between the zygotes and the false discovery rates (FDRs; q-values) were obtained. Genes with an FDR < 0.05 were extracted as DEGs.

### 4.6. Semi-Quantitative RT-PCR

The cDNAs of diploid and polyspermic zygotes at 4–5 h after fusion were synthesized as described above, and used as templates for PCR reaction. For PCR, 1 µL of the cDNA (200 pg/µL) was used as the template in a 50 µL PCR reaction with 0.3 µM of primers using KOD-FX DNA polymerase (Toyobo, Osaka, Japan) as follows: 30 or 35 cycles of 98 °C for 10 s, 55 °C for 30 s, and 68 °C for 1 min. Expression of the ubiquitin gene (Os02g0161900) was monitored as an internal control. Primer information is presented in Supplementary Table S5.

## Figures and Tables

**Table 1:** Developmental profiles of diploid and polyspermic rice zygotes.

Ploidy	Gametes Used for Fusion	No. of Zygotes Produced	No. of Zygotes That Developed to Specific Growth Stages			
			Karyogamy	Two-Celled Embryo	Globular-Like Embryo	Cell Mass
2X	Egg + Sperm	22	18	18	18	18
3X	Egg + Sperm + Sperm	34	30	19	17	17

**Table 2:** Identified genes whose expression levels were putatively up-regulated in polyspermic zygote.

Gene ID	Expression Level (Averaged TPM)		*p* Value	*q* Value	RAP-DB Description
	Diploid Zygotes	Poly spermic Zygotes			
Os01g0115600	0.0	99.8	2.17 × 10^−5^	0.01900855	Similar to LRK14
Os01g0136000	0.0	461.4	6.81 × 10^−8^	2.03 × 10^−4^	Similar to cytosolic class I small heat-shock protein HSP17.5
Os01g0149900	0.2	199.0	6.03 × 10^−6^	0.00748057	Conserved hypothetical protein
Os01g0612500	0.2	430.2	2.16 × 10^−5^	0.01900855	Phospholipase/carboxylesterase domain containing protein
Os01g0760000	0.2	137.8	5.66 × 10^−6^	0.00743199	Similar to dynein light chain
Os01g0778500	0.0	194.2	1.56 × 10^−6^	0.00268023	Similar to predicted protein
Os01g0794800	2.5	304.5	4.17 × 10^−6^	0.00564972	Similar to subtilase
Os01g0866200	8033.4	28317.5	1.08 × 10^−5^	0.01179179	Similar to histone H3
Os02g0773200	98.0	3120.0	1.39 × 10^−5^	0.01409845	UspA domain containing protein
Os03g0119900	5982.5	22237.0	6.93 × 10^−6^	0.00836748	Similar to histone H4
Os03g0217900	1420.4	14539.4	7.16 × 10^−9^	3.22 × 10^−5^	Hypothetical protein
Os03g0227800	2108.4	9163.3	3.81 × 10^−6^	0.00549041	Conserved hypothetical protein
Os03g0292100	384.8	1346.8	8.05 × 10^−5^	0.04732313	Hypothetical conserved gene
Os03g0670700	756.9	3743.1	4.96 × 10^−5^	0.03357786	Similar to glycine rich RNA binding protein
Os03g0675600	0.2	404.2	9.73 × 10^−6^	0.01086596	Similar to phytosulfokines 3 precursor
Os04g0253000	461.5	6098.4	7.25 × 10^−5^	0.04321752	Similar to histone H1
Os04g0565500	0.0	263.3	2.93 × 10^−7^	6.88 × 10^−4^	Similar to OSIGBa0158F05.8 protein
Os04g0668800	387.1	2053.6	2.68 × 10^−6^	0.00443244	Putative thiol-disulphide oxidoreductase DCC family protein
Os05g0152201	1501.4	8510.0	5.84 × 10^−6^	0.00745909	Conserved hypothetical protein
Os05g0475400	0.0	164.1	1.90 × 10^−5^	0.01728341	Similar to alanine:glyoxylate aminotransferase-like protein
Os06g0597250	7031.8	18692.8	4.36 × 10^−5^	0.03172249	Similar to B protein
Os06g0670300	0.0	140.8	5.75 × 10^−5^	0.03626026	MYB-like transcription factor
Os07g0483500	0.0	179.3	2.29 × 10^−5^	0.01934667	Similar to phosphoribosyltransferase
Os08g0388300	0.2	123.7	7.68 × 10^−6^	0.00902684	NB-ARC domain containing protein
Os08g0409900	0.2	156.2	1.63 × 10^−5^	0.01618585	Major facilitator superfamily protein
Os09g0411500	1295.0	3925.1	8.55 × 10^−5^	0.04963598	Similar to predicted protein
Os09g0433600	1651.2	5276.6	5.30 × 10^−5^	0.03536701	Similar to histone H4
Os09g0457100	0.8	184.3	4.83 × 10^−5^	0.03323256	Cytochrome P450 family protein
Os09g0483400	560.5	8011.9	1.27 × 10^−6^	0.00227203	Similar to ubiquitin/ribosomal fusion protein
Os09g0551600	8147.5	19900.5	5.76 × 10^−5^	0.03626026	Similar to HMGd1 protein
Os10g0539500	8203.4	28218.2	1.23 × 10^−6^	0.00227203	Similar to histone H4
Os11g0222800	0.0	205.4	6.49 × 10^−5^	0.04028685	Similar to LGC1
Os11g0533400	0.0	426.6	5.85 × 10^−11^	6.54 × 10^−7^	Conserved hypothetical protein
Os11g0550100	0.0	317.8	3.74 × 10^−7^	8.36 × 10^−4^	Similar to NB-ARC domain containing protein
Os12g0127200	3.2	249.1	3.96 × 10^−5^	0.02999367	Harpin-induced 1 domain containing protein
Os12g0438000	2594.6	8152.9	1.30 × 10^−5^	0.01384279	Similar to histone H2A

**Table 3:** Identified genes with putatively down-regulated expression levels in polyspermic zygotes.

Gene ID	Expression Level (Averaged TPM)		*p* Value	*q* Value	RAP-DB Description
	Diploid Zygotes	Polyspermic Zygotes			
Os01g0341200	612.4	2.2	3.77 × 10^−6^	0.005490414	Tubulin, conserved site domain containing protein
Os01g0611900	107.1	0.0	2.24 × 10^−5^	0.019231324	Pentatricopeptide repeat domain containing protein
Os01g0622033	368.3	1.8	8.79 × 10^−5^	0.049729216	Hypothetical gene
Os01g0737700	415.1	0.6	7.21 × 10^−9^	3.22 × 10^−5^	Similar to OSIGBa0101A01.4 protein
Os01g0804200	314.4	0.2	4.58 × 10^−9^	2.93 × 10^−5^	Cytochrome P450 of the CYP94 subfamily, response to wounding and salt stress
Os01g0931400	719.8	60.2	8.76 × 10^−5^	0.049729216	Thiamin pyrophosphokinase, eukaryotic domain containing protein
Os02g0281200	701.4	0.6	4.58 × 10^−12^	6.82 × 10^−8^	Similar to NBS-LRR protein
Os02g0483500	3362.8	455.8	8.09 × 10^−7^	0.001643243	Transferase family protein
Os02g0755900	194.4	0.2	4.46 × 10^−5^	0.031722495	UDP-glucuronosyl/UDP-glucosyltransferase domain containing protein
Os02g0812000	208.8	0.2	5.71 × 10^−5^	0.036260256	NAD(P)-binding domain containing protein
Os03g0299800	197.2	0.0	1.39 × 10^−5^	0.014098454	Protein of unknown function Cys-rich family protein
Os03g0321700	2104.4	182.6	7.17 × 10^−5^	0.043217519	Similar to WRKY transcription factor 55
Os04g0177300	2108.4	13.6	1.40 × 10^−10^	1.25 × 10^−6^	HIP116, Rad5p N-terminal domain containing protein
Os04g0403500	180.6	0.0	2.44 × 10^−5^	0.020228994	NAD(P)-binding domain containing protein
Os04g0503600	3609.6	179.2	3.33 × 10^−5^	0.025674616	Similar to OSIGBa0112M24.5 protein
Os04g0510600	454.0	0.0	8.17 × 10^−6^	0.009359965	Tetratricopeptide-like helical domain containing protein
Os04g0584201	168.6	0.2	4.06 × 10^−6^	0.005649724	Hypothetical protein
Os05g0164800	116.0	0.0	1.80 × 10^−5^	0.016782781	Similar to zinc transporter 6, chloroplast precursor
Os05g0203800	564.3	0.0	5.27 × 10^−8^	1.68 × 10^−4^	Transcription factor, floral organ development
Os05g0244700	1109.0	30.0	4.42 × 10^−5^	0.031722495	Aminotransferase, class IV family protein
Os05g0594200	414.3	0.2	1.82 × 10^−7^	4.78 × 10^−4^	Similar to cation/proton exchanger 1a
Os06g0520600	8126.6	1677.2	1.71 × 10^−5^	0.016639221	Similar to zinc finger CCCH type domain containing protein ZFN-like 1
Os06g0591200	180.6	0.0	4.55 × 10^−5^	0.031797243	Conserved hypothetical protein
Os07g0170000	944.2	70.8	6.90 × 10^−5^	0.042206892	Similar to Brn1-like protein
Os07g0668900	87.4	0.0	5.52 × 10^−5^	0.036260256	Similar to serine/threonine-protein kinase PBS1
Os08g0191300	4671.0	468.6	6.61 × 10^−9^	3.22 × 10^−5^	Conserved hypothetical protein
Os08g0197300	160.8	0.4	2.59 × 10^−5^	0.021001501	F-box domain, cyclin-like domain containing protein
Os08g0224700	3489.6	787.8	3.25 × 10^−5^	0.02549321	Similar to 26S proteasome subunit RPN2a
Os08g0406900	989.1	0.6	1.39 × 10^−9^	1.03 × 10^−5^	Hypothetical protein
Os09g0133600	1001.5	0.0	3.80 × 10^−20^	1.70 × 10^−15^	Fibrillin, plastoglobule (PG) formation and lipid metabolism in chloroplasts
Os09g0433650	2942.0	197.4	3.16 × 10^−5^	0.025214678	Tobacco mosaic virus coat protein family protein
Os09g0498700	241.0	1.6	1.78 × 10^−5^	0.016782781	F-box domain, cyclin-like domain containing protein
Os09g0549300	214.2	0.0	4.47 × 10^−5^	0.031722495	Flavin-containing monooxygenase FMO family protein
Os09g0552600	2250.3	264.8	1.20 × 10^−7^	3.36 × 10^−4^	RmlC-like jelly roll fold domain containing protein
Os10g0552400	1476.6	1.8	1.82 × 10^−16^	4.07 × 10^−12^	U-box E3 ubiquitin ligase
Os11g0213000	791.6	2.6	4.66 × 10^−7^	9.91 × 10^−4^	Similar to protein kinase domain containing protein, expressed
Os11g0295900	2590.4	134.4	3.26 × 10^−6^	0.005025349	AP2-transcription factor, initiation of zygotic development
Os11g0437600	612.6	0.2	1.21 × 10^−8^	4.17 × 10^−5^	Protein of unknown function DUF506, plant family protein
Os11g0619800	220.4	0.0	2.60 × 10^−7^	6.45 × 10^−4^	Kelch related domain containing protein
Os12g0135800	316.2	0.0	9.14 × 10^−9^	3.40 × 10^−5^	Alpha/beta hydrolase fold-3 domain containing protein
Os12g0268000	585.3	1.7	1.17 × 10^−6^	0.002272031	Cytochrome P450 monooxygenase, tryptamine 5-hydroxylase
Os12g0283400	1103.2	0.2	2.98 × 10^−6^	0.004756237	Pectinesterase inhibitor domain containing protein
Os12g0637100	619.8	6.4	8.01 × 10^−9^	3.25 × 10^−5^	Similar to purple acid phosphatase

## Data Availability

The transcriptome data were deposited in the DDBJ Sequence Read Archive [51] with the accession number DRA011171.

## Supplementary Materials

The following are available online at https://www.mdpi.com/2223-7747/10/2/255/s1, Table S1: Developmental profiles of diploid and polyspermic rice zygotes, Table S2: Identified genes with putatively up-regulated expression levels in polyspermic zygotes, Table S3: Identified genes with putatively down-regulated expression levels in polyspermic zygotes, Table S4: GO enrichment analysis of up-regulated genes in polyspermic zygotes, Table S5: Primers used for semi-quantitative RT-PCR.
